# The Dynamics of PKC-Induced Phosphorylation Triggered by Ca^2+^ Oscillations in Mouse Eggs

**DOI:** 10.1002/jcp.24110

**Published:** 2012-05-07

**Authors:** Jose Raul Gonzalez-Garcia, Zoltan Machaty, F Anthony Lai, Karl Swann

**Affiliations:** 1 Institute of Molecular and Experimental Medicine, School of Medicine, Cardiff UniversityCardiff, UK; 2 Department of Animal Sciences, Purdue UniversityWest Lafayette, Indiana

## Abstract

Fertilization of mammalian eggs is characterized by a series of Ca^2+^ oscillations triggered by a phospholipase C activity. These Ca^2+^ increases and the parallel generation of diacylglycerol (DAG) stimulate protein kinase C (PKC). However, the dynamics of PKC activity have not been directly measured in living eggs. Here, we have monitored the dynamics of PKC-induced phosphorylation in mouse eggs, alongside Ca^2+^ oscillations, using fluorescent C-kinase activity reporter (CKAR) probes. Ca^2+^ oscillations triggered either by sperm, phospholipase C zeta (PLCζ) or Sr^2+^ all caused repetitive increases in PKC-induced phosphorylation, as detected by CKAR in the cytoplasm or plasma membrane. The CKAR responses lasted for several minutes in both the cytoplasm and plasma membrane then returned to baseline values before subsequent Ca^2+^ transients. High frequency oscillations caused by PLCζ led to an integration of PKC-induced phosphorylation. The conventional PKC inhibitor, Gö6976, could inhibit CKAR increases in response to thapsigargin or ionomycin, but not the repetitive responses seen at fertilization. Repetitive increases in PKCδ activity were also detected during Ca^2+^ oscillations using an isoform-specific δCKAR. However, PKCδ may already be mostly active in unfertilized eggs, since phorbol esters were effective at stimulating δCKAR only after fertilization, and the PKCδ-specific inhibitor, rottlerin, decreased the CKAR signals in unfertilized eggs. These data show that PKC-induced phosphorylation outlasts each Ca^2+^ increase in mouse eggs but that signal integration only occurs at a non-physiological, high Ca^2+^ oscillation frequency. The results also suggest that Ca^2+^-induced DAG formation on intracellular membranes may stimulate PKC activity oscillations at fertilization. J. Cell. Physiol. 228: 110–119, 2013. © 2012 Wiley Periodicals, Inc.

Intracellular Ca^2+^ oscillations driven by hydrolysis of phosphatidylinositol 4,5-bisphosphate (PIP_2_) to generate inositol 1,4,5-trisphosphate (InsP_3_) and diacylglycerol (DAG) are one of the most common trans-membrane signal transduction systems used by cells (Berridge, 1993). At fertilization in mammals, the sperm causes a prolonged series of low frequency Ca^2+^ oscillations that are driven by InsP_3_ production in the unfertilized egg (Miyazaki et al., 1993; Kurokawa et al., 2004; Swann and Yu, 2008). Substantial evidence suggests that the sperm causes these Ca^2+^ oscillations by introducing a novel, sperm-specific phospholipase C enzyme isoform, phospholipase C zeta (PLCζ), into the egg after gamete membrane fusion (Saunders et al., 2002; Swann and Yu, 2008; Nomikos et al., 2012). The exclusive introduction of PLCζ into eggs by microinjecting its cognate cRNA has been shown to precisely mimic the characteristic series of sperm-initiated Ca^2+^ oscillations observed at fertilization (Cox et al., 2002; Saunders et al., 2002). These distinctive oscillations in cytosolic free Ca^2+^ appear to involve a positive feedback loop consisting of InsP_3_-induced Ca^2+^ release and Ca^2+^-dependent production of InsP_3_ by PLCζ (Swann and Yu, 2008).

The phenomenon of Ca^2+^ oscillations initiated at fertilization in mouse eggs have been shown to be the specific trigger for egg activation events, including granule exocytosis, exit from metaphase II arrest, and entry into first mitotic division (Kline and Kline, 1992). A major issue that remains unresolved is how the intrinsically repetitive nature of the sperm-activated Ca^2+^ signals is specifically transduced into downstream egg activation events. It has been suggested that the fertilized egg is able to either, integrate the total Ca^2+^ flux, or count the number of Ca^2+^ spikes, or else read the frequency of Ca^2+^ oscillations (Meyer and Stryer, 1991; Ducibella et al., 2002; Ducibella and Fissore, 2007). So far, recruitment of maternal mRNA and embryo development to term have been found to be affected by the number of Ca^2+^ transients recorded in mouse eggs (Ozil and Swann, 1995; Ducibella et al., 2002; Ozil et al., 2006). The integral of Ca^2+^ increases in the egg has also been correlated with activation rate in the mouse (Ozil et al., 2005). The main essential target for Ca^2+^ oscillations in mouse fertilization is calmodulin-dependent protein kinase II (CaMKII; Ducibella and Fissore, 2007) and assays of CaMKII at fertilization suggest that its kinase activity oscillates in near synchrony with Ca^2+^ oscillations (Markoulaki et al., 2004). However, it is not known whether protein phosphorylation driven by CaMKII responds in a manner that is able to either count or integrate Ca^2+^ oscillations.

Another protein kinase that has been shown to increase in activity at fertilization is protein kinase C (PKC; Gallicano et al., 1997; Tatone et al., 2003; Kalive et al., 2010). PKC stimulation alone is not sufficient for egg activation, but it could play a significant role since addition of the PKC activator, PMA (phorbol myristate acetate), to mouse eggs can cause activation, and the presence of pseudo-substrate inhibitors have been reported to interfere with activation at fertilization (Gallicano et al., 1993, 1997; Moses and Kline, 1995). PKC could also play an important role in causing Ca^2+^ influx at fertilization, which is important for maintaining Ca^2+^ oscillations (Halet et al., 2004). There are 10 mammalian PKC isoforms, classified into three major subfamilies (Mellor and Parker, 1998; Newton, 2003): the conventional PKCs (cPKC) α, βI, βII, and γ are stimulated by both Ca^2+^ and DAG; in contrast, novel PKCs (nPKC) δ, ε, η, and θ are regulated by DAG but are Ca^2+^-independent. Atypical PKCs (aPKC) ζ and ι/λ are neither regulated by Ca^2+^ nor by DAG. Isoforms from all three subfamilies have been found to be expressed in mammalian eggs (Jones, 1998; Luria et al., 2000; Pauken and Capco, 2000; Halet, 2004; Baluch and Capco, 2008). A specific role for PKC may have a particular relevance for eggs because PKC can act as a decoder of Ca^2+^ oscillations (Oancea and Meyer, 1998; Cullen, 2003; Violin et al., 2003). This decoding phenomenon can involve the sequential binding of Ca^2+^ and DAG to the C2 and C1 domains of cPKCs, respectively, turning the kinase into its activated state with translocation to the plasma membrane (Oancea and Meyer, 1998; Violin et al., 2003). The cPKCs, PKCα, and PKCβI translocate to the plasma membrane during fertilization in mouse eggs (Luria et al., 2000). Significantly, GFP-tagged versions of PKCα or γ were found to translocate in response to individual Ca^2+^ transients, and following decline of Ca^2+^ to basal levels, the GFP-PKCs return to the cytosol (Halet et al., 2004). Hence, PKC activation/translocation does not appear to outlast the Ca^2+^ transients, although phosphorylation events specifically induced by the activated PKC might last for longer than the Ca^2+^ transients. However, in vitro PKC kinase assays performed on egg lysates are not able to accurately monitor phosphorylation occurring in a single egg with sufficient time resolution (Gallicano et al., 1997; Tatone et al., 2003). Consequently, it remains unknown whether each cycle of PKC activity-induced phosphorylation is able to significantly outlast the duration of each Ca^2+^ transient at fertilization.

In addition to Ca^2+^-dependent cPKC, unconventional PKCs also contribute to PKC activity at fertilization. In particular, PKCδ is implicated as being the isoform responsible for a significant proportion of the biochemically measurable PKC increase occurring at fertilization (Tatone et al., 2003). PKCδ is known to be phosphorylated during oocyte maturation and then becomes dephosphorylated during the early stages of egg activation (Viveiros et al., 2001, 2003). The phosphorylation event is essential for PKCδ activation and, since PKCδ is required for oocyte maturation, it was suggested that the PKCδ phosphorylation reflects its activation state. However, up to now there have been no studies that have measured PKCδ-specific activity in eggs in real time.

PKC-induced phosphorylation has been monitored dynamically in cells using a CKAR, a probe that undergoes changes in fluorescence resonance energy transfer (FRET) in response to phosphorylation. CKAR consists of a pseudo-substrate that is specific to PKC fused to a FHA2 domain that binds phosphothreonine. This fusion protein is in turn flanked by a cyan fluorescent protein (CFP) and yellow fluorescent protein (YFP) at either end. A change in FRET between the CFP and YFP is caused by changes in CKAR conformation when the PKC-specific substrate is phosphorylated and bound by the FHA2-binding domain (Violin et al., 2003). CKAR has been shown to be subject to phosphorylation and dephosphorylation in cells (Violin et al., 2003; Gallegos et al., 2006). Myristoylated CKAR, which is targeted specifically to the plasma membrane, has been shown to undergo oscillations in FRET signal in response to Ca^2+^ transients in cell lines. The FRET response in the plasma membrane of cells was delayed with respect to Ca^2+^ transients by 10–15 sec. In contrast, the cytoplasmic CKAR did not show any oscillations in FRET signal during Ca^2+^ oscillations (Violin et al., 2003). In mouse eggs, it is unknown whether PKC activity might show a similar Ca^2+^ response pattern to that exhibited in somatic cells. Hence, in the present study, we have monitored the dynamics of PKC-induced phosphorylation during Ca^2+^ oscillations in mouse eggs using both the cytoplasmically located CKAR, and its membrane-targeted form, MyrPalm-CKAR. In addition, we monitored phosphorylation of δCKAR, which specifically responds to PKCδ activation (Kajimoto et al., 2010). Our data show that there are distinct oscillation patterns in PKC activity within the cytoplasm and the plasma membrane that occur in response to physiological Ca^2+^ transients in mouse eggs. The stimulation of PKC activity outlasts each Ca^2+^ transient by several minutes and appears to involve both cPKCs and PKCδ. Our data suggest that in mouse fertilization, the Ca^2+^ signal-induced DAG formation may play a precise role in generating oscillations in PKC activation-mediated phosphorylation.

## Materials and Methods

### Materials

Phorbol esters (PMA), PKC inhibitors (Gö6976 and rottlerin), ionophores (thapsigargin and ionomycin), and other chemicals were purchased from Sigma–Aldrich (Dorset, UK). CKAR and MyrPalm-CKAR were obtained from Addgene (http://www.addgene.org), and δCKAR was a kind gift from Alexandra Newton.

### Gamete collection and handling

MF1 female mice were super-ovulated by intraperitoneal injection of 7.5 i.u. of PMSG (pregnant mare's serum gonadotrophin; Folligon) followed 48 h later by 10 i.u. of hCG (human chorionic gonadotropin; Folligon; Saunders et al., 2002). Eggs (13–16 h post-hCG) were released from the oviduct into warmed M2 medium (Sigma, Dorset, UK). Oocytes were held in drops of M2 medium under paraffin oil in Falcon tissue culture dishes. Cumulus cells were removed by a brief exposure to hyaluronidase and the zona pellucida removed by exposure to acid Tyrode's solution (Sigma). For all fluorescence recordings, the eggs were placed in drops of HEPES-buffered KSOM (HKSOM) media (Saunders et al., 2002). For media with Sr^2+^, HKSOM media was used where the CaCl_2_ was omitted and replaced with 10 mM SrCl_2_. Spermatozoa were expelled from the cauda epididymis of male CBA/C57 mice into 1 ml of T6 medium containing 16 mg/ml BSA, and incubated under oil for 2–3 h at 37°C and 5% CO_2_ to capacitate. For in vitro fertilization (IVF) experiments, approximately 10 µl of sperm suspension was added to the dish containing the eggs.

### cRNA synthesis and microinjection

Complementary RNA (1 µg/µl) encoding CKAR, MyrPalm-CKAR (Violin et al., 2003), δCKAR (Kajimoto et al., 2010), and mouse PLCζ (Saunders et al., 2002) were synthesized and polyadenylated using mScript™ mRNA Production System (Epicentre, Calbiochem, Nottingham, UK) following the manufacturer's instructions. Microinjection of cRNA into mature mouse eggs was performed as previously described (Saunders et al., 2002), followed by a 3 h incubation at 37°C to allow the cRNA to be transcribed at detectable levels of expression.

### Measurement of CKAR and intracellular Ca^2+^

Zona-free MII eggs were kept in HKSOM under mineral oil at 37°C on the heated stage chamber of an inverted microscope (Nikon UK, Kingston upon Thames, UK). For Ca^2+^ measurements, Rhod-dextran was co-injected with CKAR or PLCζ cRNAs. One of the issues when measuring FRET together with Ca^2+^ concentration changes is that the fluorescence spectra from YFP and CFP overlap with some fluorescent Ca^2+^ indicators (e.g., Fura2, Fluo3, and FuraRed). This potential for fluorescence signal “spill-over” can distort FRET ratios. In contrast, Rhod-dextran is a long-wavelength Ca^2+^ indicator with a fluorescence excitation and emission maxima of 530 and 576 nm, respectively, and it is the dextran-coupled version of Rhod2 that is retained in the cytoplasm. Fluorescence was captured using a 20 × 0.75 NA objective at 10-sec intervals with a cooled CCD camera (Coolsnap HQ_2_, Photometrics, Tucson, AZ) and *In Vivo* software. The excitation light source was a white LED lamp (OptoLEDLite, Cairn Research Ltd., Faversham, UK). Filters (10 nm bandwidth) were controlled using filter wheels (Lambda 10-3; Sutter Instruments, Novato, CA). FRET signals were measured by taking the ratio of emission at 470 and 535 nm with excitation at 430 nm. Rhod-dextran was excited with 550 nm light and emission collected at 600 nm. A multi-band filter (XF2054, from GlenSpectra, Stanmore, UK, part of HORIBA Scientific) was placed in the dichroic filter block housing to allow excitation and emission at the selected wavelengths. Image J and SigmaPlot software (Systat Software, Inc., Hounslow, UK) were used for data analysis. Data are plotted as the ratio of cyan fluorescence to yellow emission and values normalized to the percentage change from the start of the experiment. Specific PKC inhibitors Gö6976 and rottlerin, were used to act upon conventional PKCα and PKCβ, and the novel PKCδ isoforms, respectively.

### Confocal imaging

CKAR and MyrPalm-CKAR cRNA were expressed in eggs as described above, and δCKAR was co-injected with PLCζ and incubated for 2–8 h. Single snapshots were taken of the subcellular distribution corresponding to CKAR/MyrPalm-CKAR/δCKAR FRET probes using a confocal microscope (TCS SP5; Leica, Milton Keynes, UK), under a 20× (0.75 NA) lens, and an argon laser. FRET signal was determined using the same ratiometric settings as described above and data were analyzed using Image J.

### Statistical analysis

The % CKAR FRET changes of individual signal increases were calculated based on the mean of three random spikes taken from each oscillating egg trace and divided by the total number of eggs. The “n” refers to the total number of eggs examined for each experiment type.

## Results

### Monitoring PKC-induced phosphorylation in eggs

**Fig. 1:**
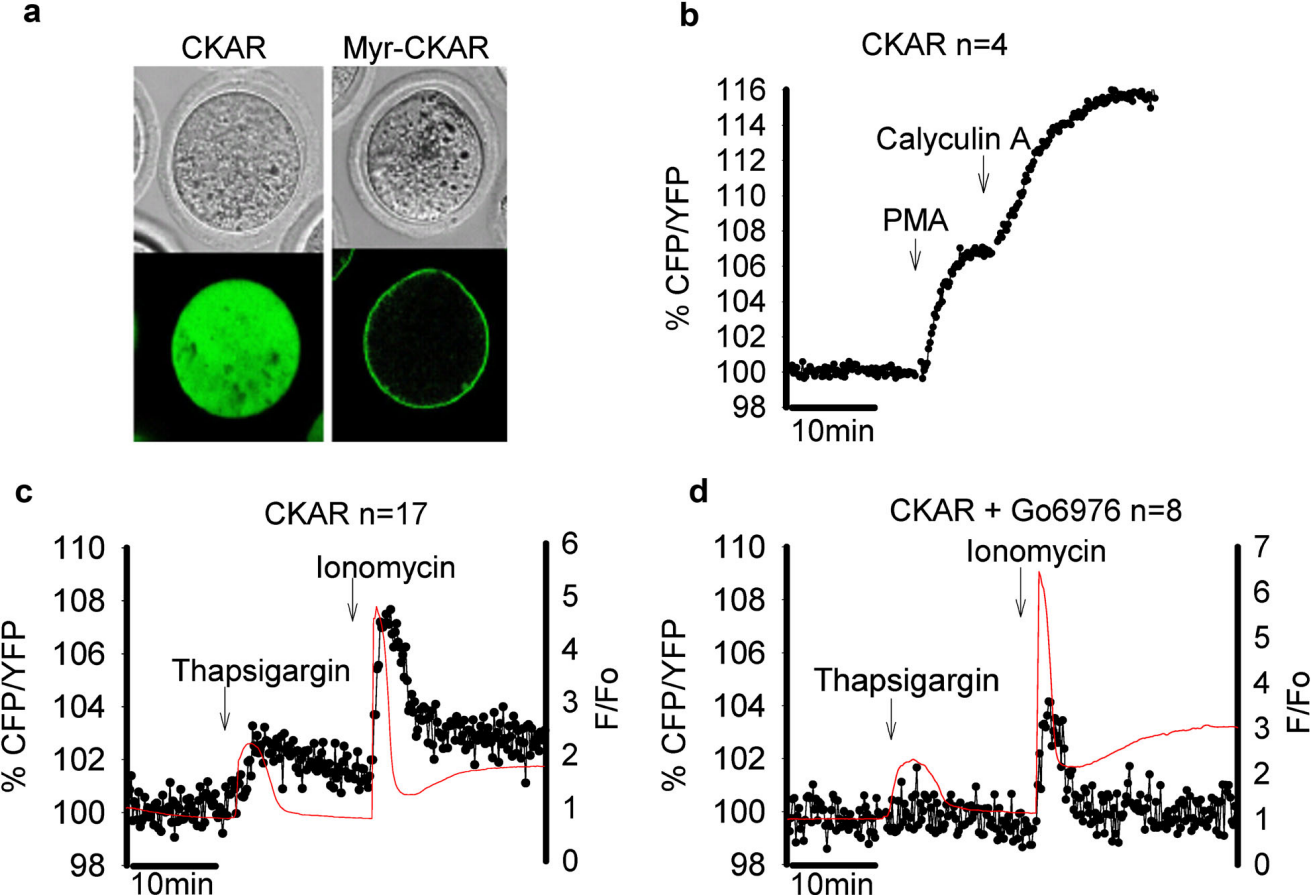
Monitoring PKC activation in mouse eggs with CKAR probes. a: Bright field (top) and confocal fluorescence (lower) images of eggs expressing cytosolic CKAR and plasma membrane-targeted MyrPalm-CKAR. In (b) the % change in CKAR FRET signal (CFP/YFP) is shown for a representative egg in response to the addition of PMA (200 nM) and calyculin A (100 nM). In (c) the CKAR ratio (black circles and lines) is plotted alongside the Ca^2+^ levels (red line) which are expressed as a ratio of Rhod-dextran fluorescence over the starting fluorescence level in eggs. Responses are shown from typical eggs in response to thapsigargin (20 µM) and then ionomycin (5 µM). In (d) the conditions are the same as in (c) but the egg was incubated in the presence of conventional PKCs inhibitor Gö6976 (10 µM) before the addition of thapsigargin and ionomycin. The “n” numbers refer to the total number of eggs examined for each experiment type. [Color figure can be seen in the online version of this article, available at http://wileyonlinelibrary.com/journal/jcp]

We found that CKAR was effectively expressed in mature mouse eggs, following microinjection of its cRNA, as indicated by fluorescence detected in the CFP and YFP channels. PKC activity, as reflected by the phosphorylation of CKAR, was monitored by measuring the ratio of the CFP to YFP signal intensity and plotted as the percentage change over the starting ratio versus time. These data are presented as the inverse of FRET efficiency, since there is an increase in this ratio with increased phosphorylation (Violin et al., 2003). Confocal images of CKAR show that it is distributed widely throughout the cytosol, with the possible exception of some exclusion by organelles (Fig. 1a). In contrast, MyrPalm-CKAR was detected specifically in the plasma membrane (Fig. 1a). This distinct localization is consistent with the fact that MyrPalm-CKAR contains seven residues of the Lyn kinase fused to the N-terminus that targets the probe to the membrane via myristoylation and palmitoylation post-translational modifications (Zacharias et al., 2002). Figure 1b shows that the addition of the potent PKC activator, PMA (200 nM), caused a CFP/YFP signal increase which reached saturation after addition of the phosphatase inhibitor, calyculin A (16.9 ± 0.25%, n = 4). Similar results were seen with MyrPalm-CKAR (data not shown). These data suggest that CKARs can be successfully expressed in mouse eggs and respond to stimuli that specifically activate PKC, and that endogenous phosphatases are continuously active in reducing the level of PKC-induced phosphorylation (Violin et al., 2003). It also reveals that the full dynamic range of the CKAR expressed in mouse eggs involves changes of <10% of the resting signal.

We tested the Ca^2+^-dependence of the CKAR response by the addition of the Ca^2+^ pump inhibitor, thapsigargin, and the Ca^2+^ ionophore, ionomycin, both of which cause monotonic Ca^2+^ increases in mouse eggs. Figure 1c shows that addition of thapsigargin triggered a CKAR signal increase of only 3.30 ± 0.84% (n = 17), whereas the subsequent addition of ionomycin effected a 7.78 ± 1.96% (n = 17) FRET increase. The greater FRET increase produced by ionomycin was correlated with a larger amplitude Ca^2+^ transient. The Ca^2+^-induced CKAR signal increase was diminished, although not abolished, in the presence of cPKC inhibitor, Gö6976 (10 µM), with either thapsigargin (1.92 ± 0.79%, n = 8) or ionomycin (4.56 ± 1.20%, n = 8; Fig. 1d). These Gö6976-mediated inhibition data suggest that there is a partial contribution of cPKC-mediated phosphorylation to the CKAR response. It should be noted that Gö6976 is the only commonly used PKC inhibitor that is non-fluorescent and does not interfere with the FRET signal. As reported previously, other broad-spectrum PKC inhibitors (e.g., Gö6983 or BIM) are fluorescent and cannot be readily used to inhibit CKAR responses without interfering with the CFP or YFP fluorescence signals required for FRET analysis (Gallegos et al., 2006).

### PKC-induced phosphorylation at fertilization

**Fig. 2:**
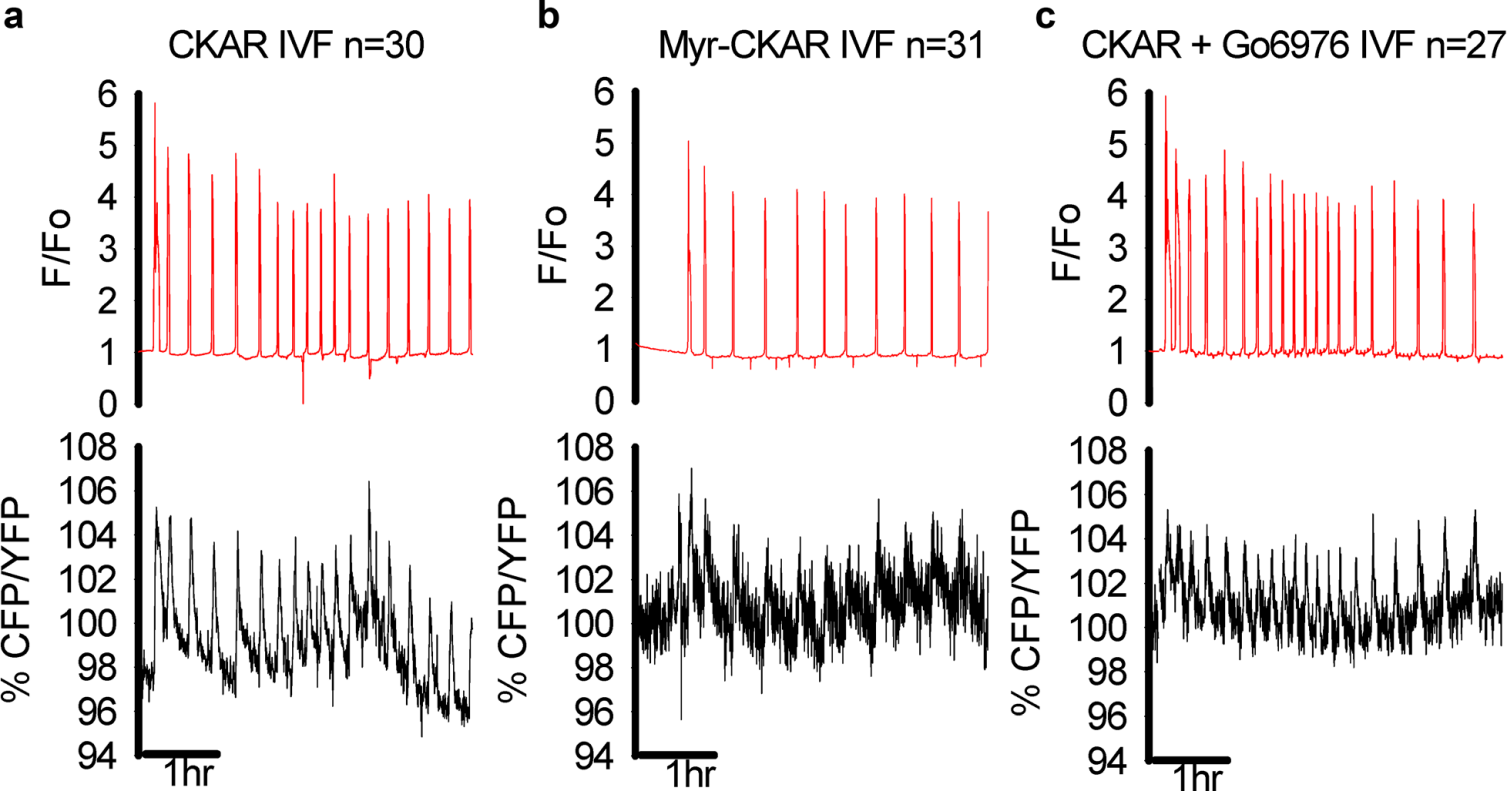
The dynamics of CKAR during fertilization of mouse eggs. The pattern of Ca^2+^ oscillations traces (top) for representative eggs that were fertilized after expressing CKAR or MyrPalm-CKAR (bottom). a, Pattern of oscillations in CKAR is shown as the % change of the CFP/YFP ratio. Eggs expressing (b) MyrPalm-CKAR and (c) CKAR, incubated in the presence of 10 µM Gö6976. As before, the “n” numbers refer to the total number of eggs examined for each experiment type. [Color figure can be seen in the online version of this article, available at http://wileyonlinelibrary.com/journal/jcp]

PKC-induced phosphorylation was monitored during IVF, using both CKAR and MyrPalm-CKAR, and their FRET signal change measured every 10 sec, alongside the occurrence of cytosolic Ca^2+^ oscillations. Figure 2a shows that Ca^2+^ oscillations following IVF of mouse eggs occurred in near synchrony with oscillatory increases in the cytoplasmic CKAR signal. The plasma membrane-localized MyrPalm-CKAR also showed comparable patterns of oscillatory FRET signal changes, similar in form to the cytoplasmic CKAR probe (Fig. 2b). The oscillatory increases in CKAR signal were small and typically displayed less than a 5% ratio change. This change was entirely due to CKAR since control IVF experiments conducted in the absence of Rhod-dextran still showed oscillatory CKAR increases, and measuring Ca^2+^ oscillations in the absence of CKAR showed no discernable oscillations in the CFP/YFP channel (Supplementary Fig. S1). The overall duration of Ca^2+^ oscillations in fertilizing eggs was not different between eggs with or without CKAR or MyrPalm-CKAR (Table 1). Hence all of the eggs studied, stopped their Ca^2+^ oscillations on schedule. Since the cessation of the Ca^2+^ signal is due to the formation of pronuclei, this suggests that the timing of egg activation events was unaffected by the presence of CKAR or MyrPalm-CKAR. In these IVF experiments, the CKAR response was not blocked by the presence of Gö6976 in fertilized eggs (Fig. 2c). Moreover, no inhibitory effect was seen following Gö6976 addition upon the CKAR oscillations induced by either Sr^2+^ or PLCζ (data not shown). Thus, it is unclear whether this oscillatory CKAR phosphorylation signal change occurring upon mouse fertilization involves the direct activation of cPKCs by each Ca^2+^ transient.

**TABLE 1:** Characteristics of Ca^2+^ oscillation patterns in fertilized eggs expressing various CKAR probes

Treatment	Total duration (min)	Total number of spikes	No. of spikes per hour
Control	256.47 ± 66.50	67.85 ± 33.81^a^	16.81 ± 10.14
CKAR	250.78 ± 62.30	87.65 ± 29.75^b^	22.22 ± 8.01
MyrCKAR	241.39 ± 81.24	61.27 ± 18.90^a^	17.52 ± 11.87

Mean and standard deviations. Different superscript letters in the same column indicate significant differences, *P* < 0.05.

**Fig. 3:**
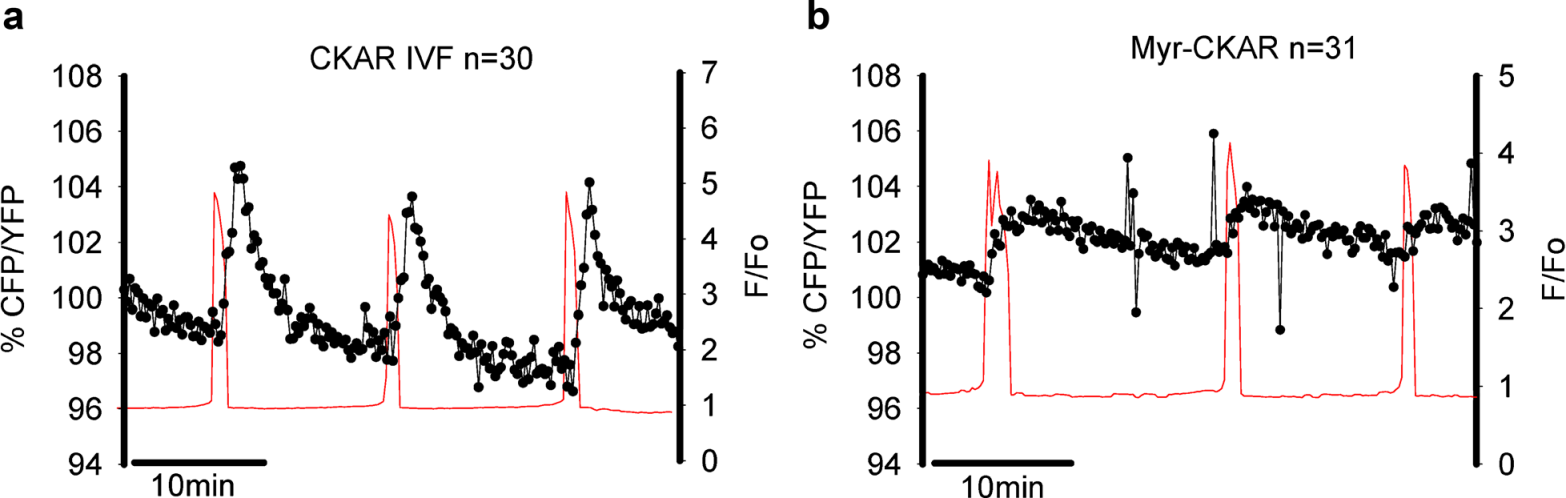
The time course of CKAR (a) or Myr CKAR (b) phosphorylation during individual Ca^2+^ transients during fertilization. The conditions and plots are the same as Figure 2 but on an expanded timescale that shows the changes in CKAR or Myr CKAR signal (black dots joined by a black line) on top of individual Ca^2+^ transients. The trace section is taken from the two typical recordings. % CKAR or Myr CKAR changes of individual phosphorylation increases were calculated based on the mean of three spikes taken from each oscillating egg trace and divided by the total number of eggs (n). [Color figure can be seen in the online version of this article, available at http://wileyonlinelibrary.com/journal/jcp]

A consistent feature of the CKAR-mediated oscillations at fertilization was that each of the FRET transients showed a different time-course relative to the Ca^2+^ transients. Figure 3 shows a series of three Ca^2+^ transients during IVF at a higher time resolution. The amplitude for the cytosolic CKAR (4.36 ± 0.53%, n = 30) was relatively larger than that for MyrPalm-CKAR (3.42 ± 0.24%, n = 31). However, for both CKAR and MyrPalm-CKAR, the peak of the FRET transient occurred 10–30 sec after the peak of the Ca^2+^ signal. In addition, the CKAR response displayed a slower decline, and did not return to baseline until ∼5 min after the Ca^2+^ transient had finished. However, it was notable that each CKAR signal increase had returned to near baseline value prior to initiation of the next Ca^2+^ spike, and hence there was no sign of progressive accumulation of the CKAR signal in Figure 2 or 3.

**Fig. 4:**
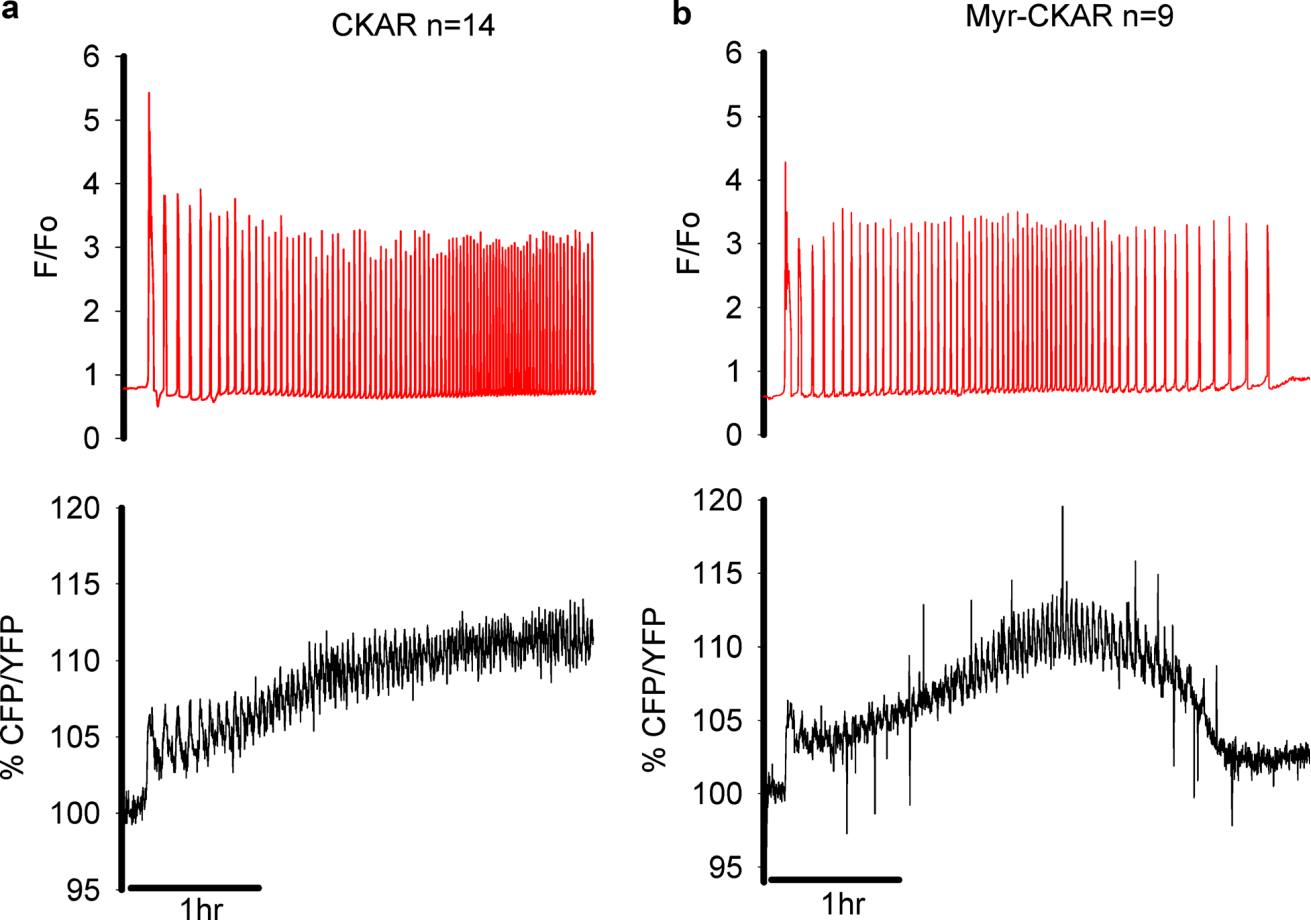
CKAR phosphorylation with high frequency Ca^2+^ oscillations. Eggs expressing (a) CKAR (n = 14) or (b) MyrPalm-CKAR (n = 9) were microinjected with a high dose of PLCζ (0.1 µg/µl pipette concentration) to trigger high frequency Ca^2+^ oscillations. The plots are in the same format as Figure 1 with Ca^2+^ as a red line and CKAR ratios as black lines. [Color figure can be seen in the online version of this article, available at http://wileyonlinelibrary.com/journal/jcp]

To determine if any long-term integration of response could occur, we tested the effects of higher frequency oscillations. Injecting high concentrations of PLCζ cRNA has been shown to cause high-frequency Ca^2+^ oscillations in mouse eggs (Saunders et al., 2002). In Figure 4, eggs were microinjected with a calibrated amount of PLCζ (0.1 µg/µl pipette concentration) that was specifically chosen to generate high-frequency Ca^2+^ oscillations. With either CKAR or MyrPalm-CKAR there was an increase in FRET signal that did not fully return to baseline between Ca^2+^ oscillations, hence the CKAR response appeared to integrate with time. However, even in these cases the FRET signal could decline considerably as the frequency of oscillations decreased (as in Fig. 4b). These data suggest that only high-frequency Ca^2+^ oscillations are able to produce a significant integration of PKC-induced phosphorylation in eggs.

### PKCδ activity in eggs

**Fig. 5:**
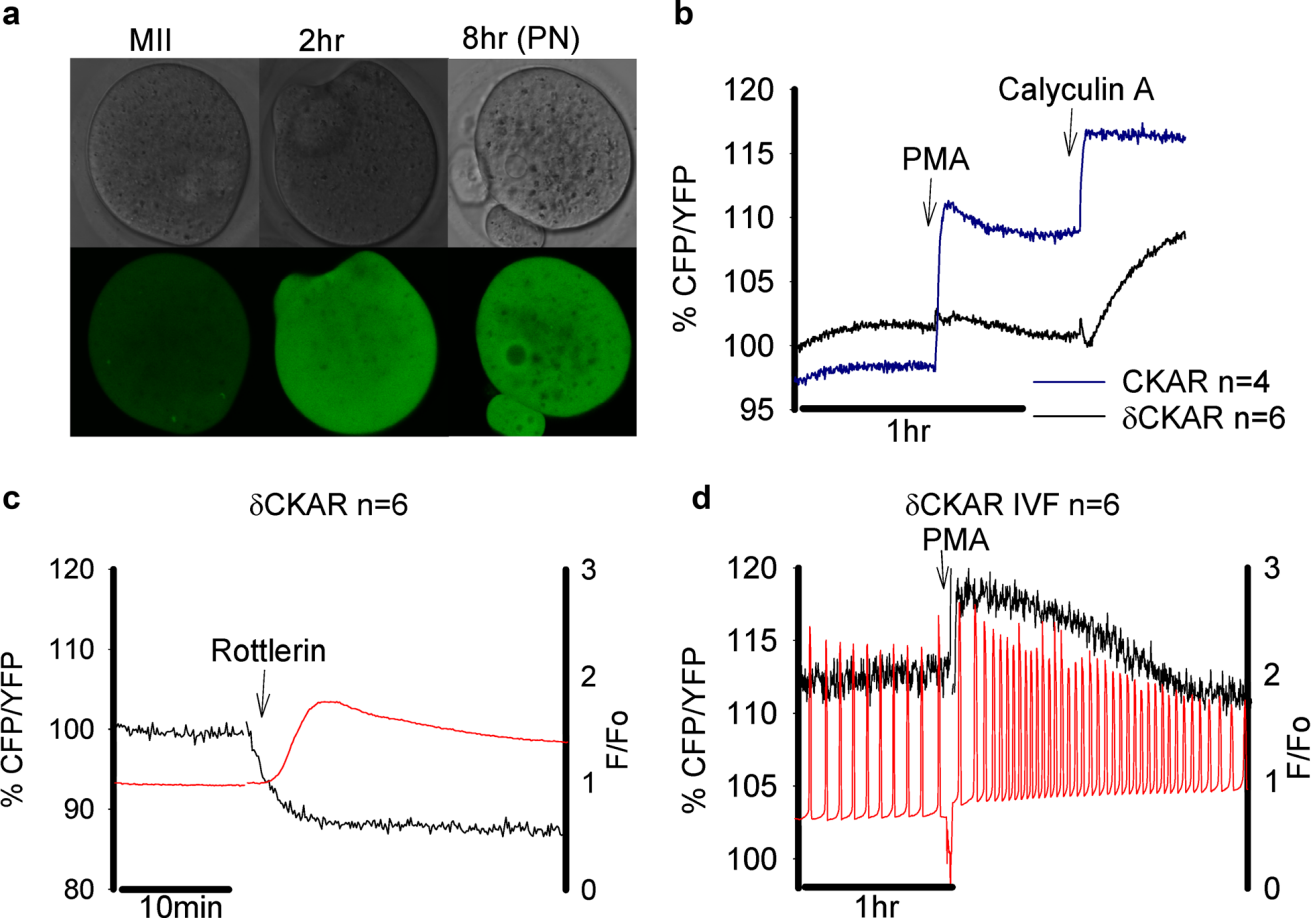
The δCKAR FRET signal and Ca^2+^ changes in mouse eggs. In (a) bright field and confocal images of eggs expressing δCKAR in unfertilized eggs, 2 h and 8 h (PN-pronucleus) after PLCζ injection. In (b) the CKAR response (blue line) and δCKAR response (solid line) are shown in an unfertilized mouse egg in response to 200 nM PMA and then 100 nM calyculin A. In (c) the δCKAR and Ca^2+^ response in an unfertilized egg is shown upon addition of rottlerin (2 µM). In (d) Ca^2+^ oscillations are shown in an egg (in red) at fertilization and the δCKAR signal is plotted (superimposed in black). Arrow shows the time, at which PMA (200 nM) was added. Sample traces are shown and the “n” numbers indicate the total number of eggs. [Color figure can be seen in the online version of this article, available at http://wileyonlinelibrary.com/journal/jcp]

Previous studies have implicated a role for PKCδ in egg activation at fertilization, so we conducted similar experiments to those described above in mouse eggs, using a newly developed PKCδ isoform-specific probe, δCKAR (Kajimoto et al., 2010). The δCKAR was expressed in mouse eggs throughout the cytoplasm, and persisted for at least 8 h after PLCζ cRNA injection, with some δCKAR signal being present in the pronucleus (Fig. 5a). There was only a very small increase in the δCKAR signal upon PMA addition (Fig. 5b; 2.04 ± 0.29%, n = 6), compared to eggs injected with conventional CKAR (11.22 ± 0.83%, n = 4). Only when we added the phosphatase inhibitor calyculin A, did the δCKAR signal show a significant response (5.13 ± 0.53%) similar to that of CKAR (5.68 ± 0.25%), although the time course was slow. These results suggest that PKCδ cannot be readily activated by PMA in mouse eggs. This could be explained if this isoform of PKC already has some activity in unfertilized mature MII mouse eggs (Viveiros et al., 2003). To test this hypothesis, rottlerin, a known PKCδ-specific inhibitor, was added to mature unfertilized mouse eggs. Figure 5c shows that this inhibitor caused a significant decrease in the δCKAR signal (12.74 ± 2.15%). Rottlerin also caused a drop in the CKAR signal in unfertilized eggs, indicating that the majority of PKC activity in mouse eggs may derive from that of PKCδ (Supplementary Fig. S2a). It was also noted that rottlerin addition caused a small Ca^2+^ increase in mouse eggs (Fig. 5c) and an immediate decrease in the oscillating CKAR signal in fertilized eggs, which was followed by a gradual increase in the cytosolic free Ca^2+^ levels and eventually, the cessation of the sperm-induced Ca^2+^ oscillation (Supplementary Fig. S2b). These changes might be due to an effect upon Ca^2+^ influx (Xu, 2007), but is unlikely to account for the decrease in δCKAR signal, since this was delayed in comparison to the rottlerin-induced FRET change. These data support the idea that PKCδ is already active to some extent in an unfertilized mouse egg. However, in contrast to the unfertilized egg, PMA caused a δCKAR signal increase in fertilizing eggs when it was added during the course of Ca^2+^ oscillations (5.93 ± 1.66%; Fig. 5d) at about 2 h after sperm addition. This PMA-induced signal increase suggests that whilst PKCδ is mostly active in unfertilized mouse eggs, it may decline in activity during the early stages of egg activation. Interestingly, there were small oscillations in the δCKAR signal at fertilization before we added PMA (Fig. 5d), which suggests that some PKCδ activity can still be further stimulated in the fertilizing mouse egg.

**Fig. 6:**
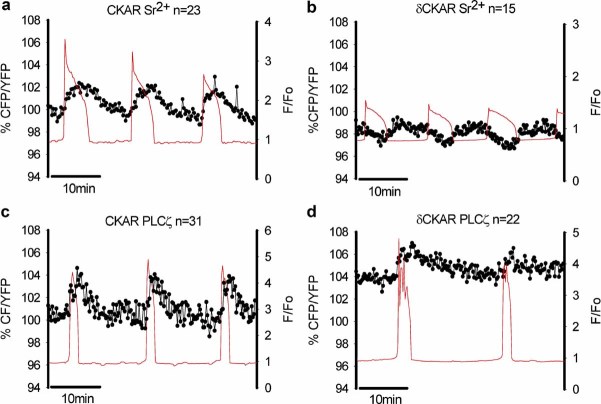
The time course of CKAR changes during Ca^2+^ oscillations in eggs. In (a,b) the eggs are undergoing Ca^2+^ oscillations in response to Sr^2+^ media, with (a) showing the CKAR signal and (b) showing the δCKAR response. In (c,d) eggs are undergoing Ca^2+^ oscillations following microinjection of PLCζ (0.01 µg/µl pipette concentration), with (c) showing the CKAR response and (d) showing the response with δCKAR. The CKAR ratio signal is shown in black (dots and lines) and the Ca^2+^ is shown in red, as before. Sample traces are shown and the “n” numbers indicate the total number of eggs. [Color figure can be seen in the online version of this article, available at http://wileyonlinelibrary.com/journal/jcp]

Since PKCδ can only be stimulated by DAG and not by Ca^2+^ directly, we examined δCKAR signals during Ca^2+^ oscillations caused by two different stimuli, Sr^2+^ or PLCζ. Figure 6 shows that both Sr^2+^- and PLCζ-induced Ca^2+^ oscillations lead to δCKAR signal increases similar in form to those seen with IVF in Figure 3. However, the δCKAR phosphorylation signal oscillations (Fig. 6b) generated by Sr^2+^ were even more delayed compared with those observed for conventional CKAR (Fig. 6a). In addition, the δCKAR signal peak occurred after the Ca^2+^ levels in the egg had declined, leading to δCKAR oscillations that were out of phase with Ca^2+^ oscillations. There is also a slight reduction in the Sr^2+^-induced signal changes with δCKAR (2.32 ± 0.22%, n = 15) compared to conventional CKAR (3.63 ± 0.59%, n = 23). In contrast, Figure 6c,d shows that PLCζ caused Ca^2+^ oscillations and similar FRET oscillatory signal increases using either cytosolic CKAR (4.97 ± 0.33%) or δCKAR (4.47 ± 0.46%). These signal changes are comparable in amplitude and pattern to those seen at fertilization. These data suggest that PKCδ responds differently to Sr^2+^ compared with fertilization by sperm or with PLCζ.

## Discussion

Mammalian fertilization is characterized by a sperm-induced series of Ca^2+^ oscillations in the egg that are critical for the physiological activation of embryo development (Kline and Kline, 1992; Ducibella et al., 2002). Previous studies have shown that PKC activity is increased at fertilization in mouse eggs (Gallicano et al., 1997; Tatone et al., 2003). There is also evidence that PKC plays a role in normal meiotic resumption after fertilization in the mouse (Gallicano et al., 1993, 1997; Moses and Kline, 1995). In this study, we have specifically set out to study the precise relationship between Ca^2+^ oscillations and PKC activity, since this topic has not been previously addressed. This is due to the absence of precise time resolution when using cell extract-based biochemical assays of PKC activity. This study shows for the first time the dynamic changes in PKC-induced phosphorylation events during fertilization in a living mammalian egg. We have achieved this by using FRET-based probes for PKC-induced phosphorylation (CKARs) to measure these dynamic changes alongside Ca^2+^ oscillations. CKAR and its subcellular-targeted derivatives have shown to be specific for monitoring PKC-induced phosphorylation and are subject to dephosphorylation by cellular phosphatases (Violin et al., 2003; Gallegos et al., 2006). The expression of CKAR or MyrPalm-CKAR did not appear to have any inhibitory effect upon egg activation since Ca^2+^ oscillations terminated similar to controls and the cessation of oscillations in mouse eggs is due to pronuclear formation (Marangos et al., 2003). Our data show that PKC-induced phosphorylation events can outlast the duration of individual Ca^2+^ spikes by several minutes. Significantly, this prolongation of phosphorylation relative to the Ca^2+^ signal can occur in both the cytoplasmic compartment and the plasma membrane. Furthermore, experiments using the PKCδ isoform-specific probe, δCKAR (Kajimoto et al., 2010), suggest that Ca^2+^ can stimulate PKCs both through the generation of DAG, as well as via direct Ca^2+^-dependent binding and activation.

### Integration of Ca^2+^ oscillations by PKC-induced phosphorylation

One of the outstanding Ca^2+^ signaling issues in eggs is how oscillatory Ca^2+^ changes are translated, and possibly integrated, into changes in the activity of relevant target enzymes (Ducibella and Fissore, 2007). Previous studies in somatic cell lines have found that cPKC, such as PKCγ, can act as an integration module for decoding Ca^2+^ oscillations that are associated with phosphoinositide turnover, by virtue of its ability to become activated and translocate to the plasma membrane (Oancea and Meyer, 1998). This integration of Ca^2+^ oscillations relies upon DAG production within the plasma membrane causing prolonged membrane residence of cPKC. Fertilization also stimulates translocation of PKCα, β, and γ to the plasma membrane (Raz et al., 1998; Luria et al., 2000; Baluch et al., 2004). At fertilization, however, whilst each Ca^2+^ transient leads to plasma membrane translocation of cPKC-GFP in mouse eggs, the cPKC-GFPs return to the cytoplasm within ∼10 sec of the cytosolic-free Ca^2+^ returning to resting levels (Halet et al., 2004). This implies only a very limited integration of PKC activity, which could be due to very limited accumulation of DAG in the plasma membrane during fertilization (Halet et al., 2004; Yu et al., 2008). Nevertheless, a PKC signal in the cell may persist for a longer period because phosphorylated substrates may outlast the PKC translocation process.

The dynamics of phosphorylation events induced by PKCs has been monitored in somatic cell lines using CKAR, and derivatives of CKAR targeted to sub-cellular compartments (Cullen, 2003; Violin et al., 2003). It is not known whether the phosphorylation of CKAR precisely reflects the phosphorylation and dephosphorylation of endogenous PKC substrates. However, CKAR has been shown to be a specific substrate for PKC and subject to dephosphorylation by the same type of phosphatases as endogenous PKC substrates. It is important to note that the increase in CKAR signal we see at fertilization is relatively small (∼5%), but this reflects the intrinsic limitation of the probe, rather than the response of the cell, which is likely to involve a much larger change in phosphorylation. In fact FRET probes of the same class as CKAR all show small changes in signal. In a previous study in eggs, for example, a CFP/YFP-based probe for InsP_3_ showed <5% increases in eggs after PLCζ injection, despite the fact that InsP_3_ probably increases by several fold (Shirakawa et al., 2006).

In the study in somatic cells by Violin et al. (2003), it was found that phosphorylation of plasma membrane CKAR outlasted the cytoplasmic Ca^2+^ spikes by 10–15 sec. Whilst significant in cell lines, this degree of integration would not be sufficient in mammalian fertilized eggs, where each Ca^2+^ transient lasts for approximately 1 min and are typically spaced 10 min apart. We found that the phosphorylation of CKAR in both the cytoplasm and plasma membrane is maintained for about 5 min after each Ca^2+^ transient during repetitive oscillations in mouse eggs, which is significantly longer than the 10–15 sec observed in previous somatic cell studies using CKAR. However, this extended phosphorylation time-course still results in the PKC signal in eggs returning to near-resting levels within the 10 min before the next Ca^2+^ transient begins. This response profile might be sufficient for the PKC-stimulated Ca^2+^ influx that occurs after each Ca^2+^ transient in mouse eggs (McGuinness et al., 1996), but it does not provide the basis for explaining longer-term effects. In contrast, we were able to see a clear accumulation of the CKAR response when we injected high concentrations of PLCζ to deliberately cause high-frequency Ca^2+^ oscillations. This result suggests that the degree of CKAR phosphorylation can be varied in response to the frequency of Ca^2+^ oscillations. However, this cumulative effect is only observed with a Ca^2+^ oscillation frequency well above that observed physiologically at fertilization. Hence, it appears unlikely that the primary phosphorylation events induced by PKC activation are able to integrate the lower frequency Ca^2+^ oscillations occurring during normal fertilization.

### Ca^2+^-induced DAG formation as the stimulus for PKC

In our experiments, there is a distinct increase in the CKAR signal observed in response to each Ca^2+^ transient. The elevations in free Ca^2+^ concentration could stimulate this PKC activity increase by two potential mechanisms; by direct binding of Ca^2+^ to the C2 domain or by stimulating PLC-mediated DAG production, which then binds to the PKC C1 domain. Ca^2+^-stimulated DAG production is likely to occur in fertilizing mammalian eggs because it has been shown that sperm PLCζ activity is very sensitive to increases in cytosolic Ca^2+^ levels (Nomikos et al., 2005). In addition, Ca^2+^-dependent InsP_3_ production has been shown to be part of the mechanism of Ca^2+^ oscillations and this implies that oscillatory increases in both InsP_3_ and DAG occur during each Ca^2+^ transient (Swann and Yu, 2008). Our data suggest that PKCs may be stimulated directly by Ca^2+^, but that Ca^2+^-induced DAG formation may also form a significant component of the PKC response. All of the stimuli that cause an elevation of Ca^2+^ in eggs lead to an increase in the CKAR signal. The CKAR response was not effectively blocked by the cPKC inhibitor, Gö6976, with the exception of thapsigargin, which only causes a small increase in Ca^2+^. Surprisingly, we found no effect of Gö6976 on fertilization-induced CKAR increases. Either Gö6976 may not be fully effective at inhibiting PKC in mouse eggs, or it could also suggest that the conventional isoforms of PKC are partially involved in stimulating some of the CKAR in response to Ca^2+^ elevation. This second idea is further supported by the finding, that despite its pre-existing basal activity in unfertilized eggs, δCKAR can be further stimulated by the Ca^2+^ transients induced by fertilization, PLCζ and Sr^2+^-containing media. The presumed mechanism for Ca^2+^ to stimulate δCKAR is via DAG production. Sr^2+^ media is of particular interest because it is thought to act via stimulating InsP_3_ receptors to release Ca^2+^ (Marshall and Taylor, 1994; Zhang et al., 2005). Unlike the sperm and PLCζ, Sr^2+^ medium does not lead to any detectable down-regulation of InsP_3_ receptors and so is not expected to cause significant PIP_2_ hydrolysis (Brind et al., 2000; Jellerette et al., 2000). Our data show that Sr^2+^-induced Ca^2+^ oscillations are accompanied by some δCKAR signal, implying that these Ca^2+^ increases alone can cause some DAG production. This Sr^2+^-mediated mechanism could involve Ca^2+^ stimulation of other egg-derived PLCs such as PLCβ1, which appears to be stimulated to some extent at fertilization in mouse eggs (Igarashi et al., 2007). It was, however, noted that the amplitude and time course of δCKAR stimulation was different between Sr^2+^ and PLCζ. The Sr^2+^ response was smaller and more delayed with respect to the Ca^2+^ transient than that with PLCζ, which, in turn, could be due to a delay in DAG production. Previous studies have found that Ca^2+^ ionophores induced DAG accumulation in the plasma membrane with a delay of a few minutes in unfertilized eggs (Halet et al., 2004; Yu et al., 2008). This implies that the Ca^2+^-induced stimulation of PLCζ generates DAG much more rapidly than that provided by Ca^2+^ stimulation of other egg-derived PLCs.

### Basal PKC activity in eggs

Previous studies have suggested that there might be a basal level of PKC activity present in mouse eggs or muscle cells (Nicolas et al., 1998; Akabane et al., 2007). PKCδ has been shown to be phosphorylated at an activating residue in mature mouse eggs, and hence PKCδ may already be active at the MII stage (Viveiros et al., 2003). Our data are consistent with this idea, since the PKCδ-specific inhibitor, rottlerin, caused a clear decrease in the δCKAR signal in an unfertilized egg. Addition of PMA only caused a minimal increase, although there was a large increase in the δCKAR signal when added over an hour into the activation process. This result is consistent with previous reports showing that PKCδ dephosphorylation occurs after egg activation (Viveiros et al., 2003). Nevertheless, there were still small increases in the δCKAR signal associated with Ca^2+^ transients at fertilization, suggesting that PKCδ substrates are not completely phosphorylated in an unfertilized egg.

### The nature of cytoplasmic PKC activity oscillations

One of the most remarkable results of the current study was that a PKC-induced response is detected with both the plasma membrane-targeted and cytoplasmic CKAR. Previous studies of PKC in live somatic cells have shown that agonists can lead to DAG production, although PKC oscillations only occur in the plasma membrane (Oancea and Meyer, 1998; Violin et al., 2003). To date, the evidence for a PKC-induced phosphorylation response that outlasts oscillating Ca^2+^ transients (by ∼15 sec) is within the plasma membrane (Violin et al., 2003). The previous dynamic PKC imaging in mouse eggs has also entirely concerned short-term translocation to the plasma membrane (Halet et al., 2004; Yu et al., 2008). Our new data show that longer-lasting phosphorylation increases occur in fertilizing mouse eggs, both in the cytoplasm and the plasma membrane. In fact, the CKAR signal is notably stronger in the cytoplasm than at the plasma membrane. This suggests that the majority of DAG formation and subsequent PKC stimulation occurs at sites within the egg cytoplasm in response to Ca^2+^ transients. In somatic cells, agonist stimulation can lead to DAG generation in the Golgi membranes as well as the plasma membrane (Gallegos et al., 2006). Internal membrane organelles in mouse eggs could therefore also be a potential source of DAG at fertilization. In accord with this possibility, we have recently found that mouse eggs contain a significant amount of PIP_2_ specifically located in internal vesicles (Yu et al., 2012). Moreover, these discrete intracellular vesicles appear to be the precise target of PLCζ-induced PIP_2_ hydrolysis. Therefore, it is distinctly possible that the sperm-delivered PLCζ enables Ca^2+^-dependent DAG formation on intracellular PIP_2_-containing vesicles, facilitating repetitive PKC stimulation throughout the egg cytoplasm. Further experiments could address this possibility by the use of DAG-specific probes targeted to intracellular vesicles.

## References

[b1] Akabane H, Fan J, Zheng X, Zhu GZ (2007). Protein kinase C activity in mouse eggs regulates gamete membrane interaction. Mol Reprod Dev.

[b2] Baluch DP, Capco DG (2008). GSK3beta mediates a centromeric spindle stabilization by activated PKCzeta. Dev Biol.

[b3] Baluch DP, Koeneman BA, Hatch KR, McGaughey RW, Capco DG (2004). PKC isotypes in post-activated and fertilized mouse eggs: Association with the meiotic spindle. Dev Biol.

[b4] Berridge MJ (1993). Inositol trisphosphate and calcium signalling. Nature.

[b5] Brind S, Swann K, Carroll J (2000). Inositol 1,4,5-trisphosphate receptors are downregulated in mouse oocytes in response to sperm and adenophostin A but not to increase in intracellular Ca^2+^ or egg activation. Dev Biol.

[b6] Cox LJ, Larman MG, Saunders CM, Hashimoto K, Swann K, Lai FA (2002). Sperm phospholipase Czeta from humans and cynomolgus monkeys triggers Ca^2+^ oscillations, activation and development of mouse oocytes. Reproduction.

[b7] Cullen PJ (2003). Calcium signalling: The ups and downs of protein kinase C. Curr Biol.

[b8] Ducibella T, Fissore RA (2007). The roles of Ca^2+^, downstream protein kinases, and oscillatory signalling in regulating fertilization and the activation of development. Dev Biol.

[b9] Ducibella T, Huneau D, Angelichio E, Xu Z, Schultz RM, Kopf GS, Fissore R, Madoux S, Ozil JP (2002). Egg-to-embryo transition is driven by differential responses to Ca^2+^ oscillation number. Dev Biol.

[b10] Gallegos LL, Kunkel MT, Newton AC (2006). Targeted protein kinase C activity reporter to discrete intracellular regions reveals spatiotemporal differences in agonist-dependent signalling. J Biol Chem.

[b11] Gallicano GI, Schwarz SM, McGaughey RW, Capco DG (1993). Protein kinase C, a pivotal regulator of hamster egg activation, functions after elevation of intracellular free calcium. Dev Biol.

[b12] Gallicano GI, McGaughey RW, Capco DG (1997). Activation of protein kinase C after fertilization is required for remodelling in the mouse egg into the zygote. Mol Reprod Dev.

[b13] Halet G (2004). PKC signaling at fertilization in mammalian eggs. Biochem Biophys Acta.

[b14] Halet G, Tunwell R, Parkinson SJ, Carroll J (2004). Conventional PKCs regulate the temporal pattern of Ca^2+^ oscillations at fertilisation in mouse eggs. J Cell Biol.

[b15] Igarashi H, Knott JG, Schultz RM, Williams CJ (2007). Alterations of PLC β1 in mouse eggs changes calcium oscillatory behavior following fertilization. Dev Biol.

[b16] Jellerette T, He CL, Wu H, Parys JB, Fissore RA (2000). Down-regulation of the inositol 1,4,5-trisphosphate receptor in mouse eggs following fertilization or parthenogeneticactivation. Dev Biol.

[b17] Jones KT (1998). Protein kinase C at fertilization: Overstated or undervalued. Rev Reprod.

[b18] Kajimoto T, Sawamura S, Tohyama Y, Mori Y, Newton AC (2010). Protein kinase C δ-specific activity reporter reveals agonist-evoked nuclear activity controlled by Src family of kinases. J Biol Chem.

[b19] Kalive M, Faust JJ, Koeneman BA, Capco DG (2010). Involvement of the PKC family in regulation of early development. Mol Reprod Dev.

[b20] Kline D, Kline T (1992). Repetitive calcium transients and the role of calcium in exocytosis and cell cycle activation in the mouse egg. Dev Biol.

[b21] Kurokawa M, Sato K, Fissore RA (2004). Mammalian fertilization: From sperm factor to phospholipase Cζ. Biol Cell.

[b22] Luria A, Tennenbaum T, Sun QY, Rubinstein S, Breitbart H (2000). Differential localization of conventional protein kinase C isoforms during mouse oocyte development. Biol Reprod.

[b23] Marangos P, Fitzharris G, Carroll J (2003). Ca^2+^ oscillations at fertilization in mammals are regulated by the formation of pronuclei. Development.

[b24] Markoulaki S, Matson S, Ducibella T (2004). Fertilization stimulates long-lasting oscillations of CaMKII activity in mouse eggs. Dev Biol.

[b25] Marshall IC, Taylor CW (1994). Two binding sites mediate the interconversion of liver inositol 1,4,5-trisphosphate receptors between three conformational states. Biochem J.

[b26] McGuinness OM, Moreton RB, Johnson MH, Berridge MJ (1996). A direct measurement of increased divalent cation influx in fertilized mouse oocytes. Development.

[b27] Mellor H, Parker PJ (1998). The extended protein kinase C superfamily. Biochem J.

[b28] Meyer T, Stryer L (1991). Calcium spiking. Ann Rev Biophys Biochem.

[b29] Miyazaki S, Shirakawa H, Nakada K, Honda Y (1993). Essential role of the inositol 1,4,5-trisphosphate/Ca^2+^ release channel in Ca^2+^ waves and Ca^2+^ oscillations at fertilization of mammalian eggs. Dev Biol.

[b30] Moses RM, Kline D (1995). Calcium-independent, meiotic spindle-dependent metaphase-to-interphase transition in phorbol ester-treated mouse eggs. Dev Biol.

[b31] Newton AC (2003). Regulation of the ABC kinases by phosphorylation: Protein kinase C as a paradigm. Biochem J.

[b32] Nicolas JM, Renard-Rooney DC, Thomas AP (1998). Protein kinase C activity in isolated cardiac myocytes. J Mol Cell Cardiol.

[b33] Nomikos M, Blayney LM, Larman MG, Campbell K, Rossbach A, Saunders CM, Swann K, Lai FA (2005). Role of phospholipase C-ζ domains in Ca^2+^-dependent phosphatidylinositol 4,5-bisphosphate hydrolysis and cytoplasmic Ca^2+^ oscillations. J Biol Chem.

[b34] Nomikos M, Swann K, Lai FA (2012). Starting a new life: Sperm PLC-zeta mobilizes the Ca^2+^ signal that induces egg activation and embryo development: An essential phospholipase C with implications for male infertility. Bioessays.

[b35] Oancea E, Meyer T (1998). Protein kinase C as a molecular machine for decoding calcium and diacylglycerol signals. Cell.

[b36] Ozil JP, Swann K (1995). Stimulation of repetitive calcium transients in mouse eggs. J Physiol.

[b37] Ozil JP, Markoulaki S, Toth S, Matson S, Banrezes B, Knott JG, Schultz RM, Huneau D, Ducibella T (2005). Egg activation events are regulated by the duration of a sustained [Ca^2+^]_cyt_ signal in the mouse. Dev Biol.

[b38] Ozil JP, Banrezes B, Toth S, Pan H, Schultz RM (2006). Ca^2+^ oscillatory pattern in fertilized mouse eggs affects gene expression and development to term. Dev Biol.

[b39] Pauken CM, Capco DG (2000). The expression and stage specific localization of protein kinase C isotypes during mouse preimplantation development. Dev Biol.

[b40] Raz T, Ben-Yosef D, Shalgi R (1998). Segregation of the pathways leading to cortical reaction and cell cycle activation in the rat egg. Biol Reprod.

[b41] Saunders CM, Larman MG, Parrington J, Cox LJ, Royse J, Blayney LM, Swann K, Lai FA (2002). PLCζ: A sperm-specific trigger of Ca^2+^ oscillations in eggs and embryo development. Development.

[b42] Shirakawa H, Ito M, Sato M, Umezawa Y, Miyazaki S (2006). Measurement of intracellular IP_3_ during Ca^2+^ oscillations in mouse eggs with a GFP-based FRET probe. Biochem Biophys Res Comm.

[b43] Swann K, Yu Y (2008). The dynamics of Ca^2+^ oscillations that activate mammalian eggs. Int J Dev Biol.

[b44] Tatone C, DelleMonarche S, Francione A, Gioia L, Barboni B, Colonna R (2003). Ca^2+^-independent protein kinase C signalling in mouse eggs during the early phases of fertilization. Int J Dev Biol.

[b45] Violin JD, Zhang J, Tsien RY, Newton AC (2003). A genetically encoded fluorescent reporter reveals oscillatory phosphorylation by protein kinase C. J Cell Biol.

[b46] Viveiros MM, Hirao Y, Eppig JJ (2001). Evidence that protein kinase C (PKC) participates in the meiosis I to meiosis II transition in mouse oocytes. Dev Biol.

[b47] Viveiros MM, O'Brien M, Wigglesworth K, Eppig JJ (2003). Characterization of protein kinase C-δ in mouse oocytes throughout meiotic maturation and following egg activation. Biol Reprod.

[b48] Xu SZ (2007). Rottlerin induces calcium influx and protein degradation in cultured lenses independent of effects on protein kinase Cδ. Basic Clin Pharmacol Toxicol.

[b49] Yu Y, Halet G, Lai FA, Swann K (2008). The regulation of diacylglycerol production and protein kinase C stimulation during sperm and PLCζ-mediated mouse egg activation. Biol Cell.

[b50] Yu Y, Nomikos M, Theodoridou M, Nounesis G, Lai FA, Swann K (2012). PLCζ causes Ca^2+^ oscillations in mouse eggs by targeting intracellular and not plasma membrane PI(4,5)P_2_. Mol Biol Cell.

[b51] Zacharias DA, Violin JD, Newton AC, Tsien RY (2002). Partitioning of lipid-modified monomeric GFPs into membrane microdomains of live cells. Science.

[b52] Zhang D, Pan L, Yang LH, He XK, Huang XY, Sun FZ (2005). Strontium promotes calcium oscillations in mouse meiotic oocytes and early embryos through InsP3 receptors, and requires activation of phospholipase and synergistic action of InsP3. Hum Reprod.

