# Supplementary figures and images for: The Dynamics of PKC-Induced Phosphorylation Triggered by Ca2+ Oscillations in Mouse Eggs
Source: J Cell Physiol. 2012 May 7;228(1):110–9. doi: 10.1002/jcp.24110 (PMC3746124; doi:10.1002/jcp.24110)

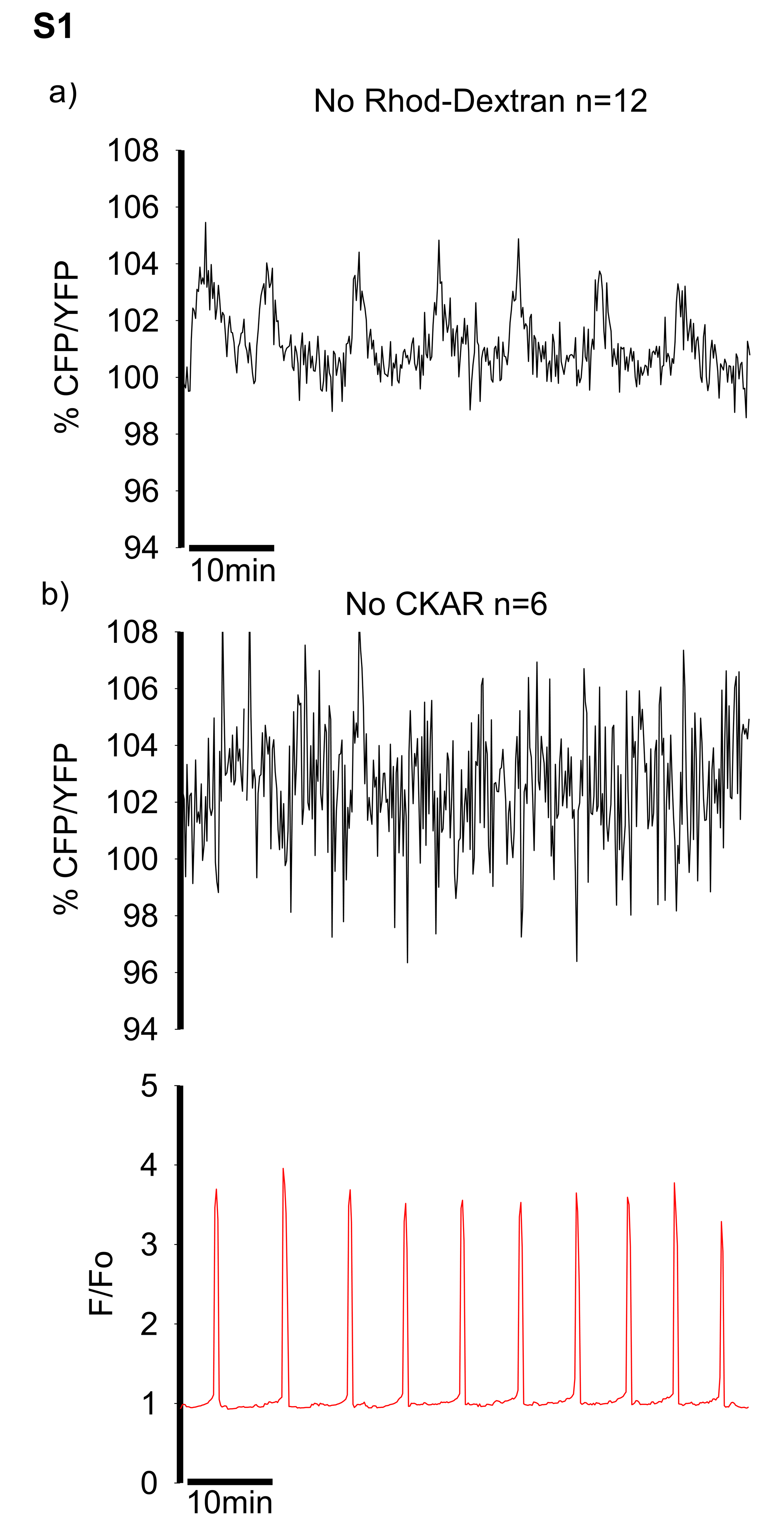

Supplement: Supplementary file 1 [file jcp0228-0110-SD1.tif]

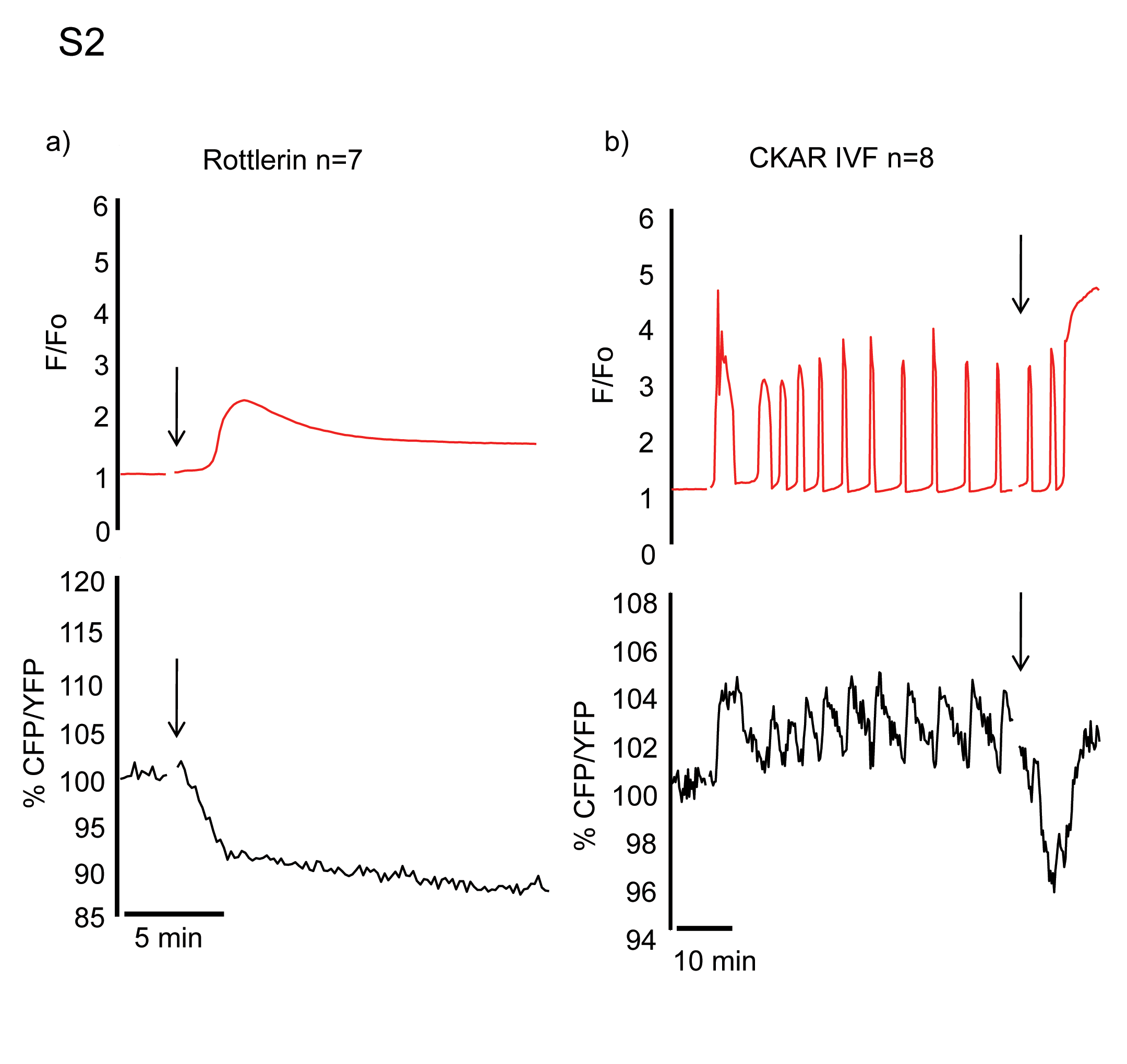

Supplement: Supplementary file 2 [file jcp0228-0110-SD2.tif]
