# Supplementary material for: The Dynamics of PKC-Induced Phosphorylation Triggered by Ca2+ Oscillations in Mouse Eggs
Source: J Cell Physiol. 2012 May 7;228(1):110–9. doi: 10.1002/jcp.24110 (PMC3746124; doi:10.1002/jcp.24110)
Supplement: Supplementary file 3 [file jcp0228-0110-SD3.doc]

**Supplementary data**

**S1** Control experiments for CKAR fluorescence changes in mouse eggs. In a) a sample trace (n=12) is shown for an egg expressing CKAR in the absence of Rhod-dextran. The recording is taken after the addition of sperm and oscillations in the CKAR signal (CFP/YFP fluorescence excitation) can be seen. At the end of the recording the egg was verified to be fertilized as indicated by two pronuclei. In b) a control egg injected with Rhod-dextran in the absence of CKAR and Ca^2+^ oscillations recorded during fertilization (n=6). The CFP/YFP fluorescence excitation signal plot is shown for a sample egg and it shows no sign of oscillations in synchrony with Ca^2+^ spikes. Note that the trace is noisier than in the presence of CKAR, since ratiometric measurements are taken from very low level of background fluorescence signals.

**S2** Effect of rottlerin, a PKCδ inhibitor, on the intracellular free Ca^2+^ level and PKC activity in unfertilized and fertilized mouse eggs. In a) unfertilized eggs were injected with Rhod-dextran and cRNA encoding CKAR. Four hours later fluorescence recordings began and after a short baseline measurement rottlerin was added to the eggs. In b) after the initiation of the fluorescence recording the injected eggs were inseminated and then rottlerin was added to the medium. The arrows indicate the addition of rottlerin (final concetration 2 μM).
